# Neuroimaging and DNA Methylation: An Innovative Approach to Study the Effects of Early Life Stress on Developmental Plasticity

**DOI:** 10.3389/fpsyg.2021.672786

**Published:** 2021-05-17

**Authors:** Isabella Lucia Chiara Mariani Wigley, Eleonora Mascheroni, Denis Peruzzo, Roberto Giorda, Sabrina Bonichini, Rosario Montirosso

**Affiliations:** ^1^Department of Developmental and Social Psychology, University of Padua, Padua, Italy; ^2^0-3 Center for the At-Risk Infant, Scientific Institute, IRCCS Eugenio Medea, Bosisio Parini, Italy; ^3^Neuroimaging Lab, Scientific Institute, IRCCS Eugenio Medea, Bosisio Parini, Italy; ^4^Molecular Biology Laboratory, Scientific Institute, IRCCS Eugenio Medea, Bosisio Parini, Italy

**Keywords:** DNA methylation, developmental plasticity, neuroimaging, early life stress, neuroimaging epigenetics

## Abstract

DNA methylation plays a key role in neural cell fate and provides a molecular link between early life stress and later-life behavioral phenotypes. Here, studies that combine neuroimaging methods and DNA methylation analysis in pediatric population with a history of adverse experiences were systematically reviewed focusing on: targeted genes and neural correlates; statistical models used to examine the link between DNA methylation and neuroimaging data also considering early life stress and behavioral outcomes. We identified 8 studies that report associations between DNA methylation and brain structure/functions in infants, school age children and adolescents faced with early life stress condition (e.g., preterm birth, childhood maltreatment, low socioeconomic status, and less-than optimal caregiving). Results showed that several genes were investigated (e.g., *OXTR*, *SLC6A4*, *FKBP5*, and *BDNF*) and different neuroimaging techniques were performed (MRI and *f*-NIRS). Statistical model used ranged from correlational to more complex moderated mediation models. Most of the studies (*n* = 5) considered DNA methylation and neural correlates as mediators in the relationship between early life stress and behavioral phenotypes. Understanding what role DNA methylation and neural correlates play in interaction with early life stress and behavioral outcomes is crucial to promote theory-driven studies as the future direction of this research fields.

## Introduction

Developmental plasticity represents an adaptive process in human and non-human animals by which a specific genotype could give rise to different phenotypes according to the experiences they live trough (Hochberg et al., 2011). This process may be described at different levels of organization arranged hierarchically from the molecular to the neural and ultimately to the behavioral level (LaFreniere and MacDonald, 2013). At the neural level, an increasing amount of neuroimaging studies is highlighting that early life stresses are correlated with changes in many brain features including morphological (i.e., cortical thickness, white matter volume, structural connectivity, etc.) and functional modifications (i.e., neural activity, functional connectivity, etc.) (Brown et al., 2014; Scheinost et al., 2017; Suppiej et al., 2017). Remarkably, existing evidences suggest that epigenetic mechanisms–such as DNA methylation–might, at least partially, explain how early life stress (i.e., prematurity, low socioeconomic status, and less-than optimal caregiving) can interact with both molecular and neural development, contributing to define behavioral outcomes during periods of developmental plasticity (Fagiolini et al., 2009).

Interestingly, a relatively new approach is exploring the possibility to combine the contributes of DNA methylation analysis and neuroimaging methods in order to better examine how the interplay between experience and genetic programs can sculpt neuronal circuits during early brain development (Lancaster et al., 2018). Looking at developmental plasticity from this combined point of view might lead to new insights into the mechanisms through which early life stresses are embedded in later-life behavioral phenotypes (Provenzi et al., 2016; Jiang et al., 2018).

As interest in this field is rapidly growing (Wheater et al., 2020), the aim of the present work is to highlight studies that take into account DNA methylation analysis and brain data in pediatric populations faced with an early life stress condition. Specifically, the present systematic review was designed at providing: (a) a preliminary account of neuroimaging and DNA methylation studies’ state of the art; (b) how different studies examined the link between DNA methylation and neuroimaging data, also considering early life stress condition and, if any, their relationship with behavior outcomes; (c) future directions of research in this field.

### DNA Methylation

Gene expression is determined not only by the DNA sequence itself and its regulatory factors but also by changes in the way the DNA is modified and packaged within the chromatin (Ozanne and Constância, 2007). The maintenance of gene expression patterns seems to be strongly related to the *tagging* on DNA and histones by enzymatic binding of chemical tags (Keverne and Curley, 2008). The set of chemical tags is called *epigenome* and, unlike the underlying genome, it could be dynamically altered by environmental conditions (Kanherkar et al., 2014). DNA methylation is one of the epigenetic mechanisms that, at least partially, mediate the gene-environment interaction (for a more comprehensive view of epigenetic mechanisms, see Giorda, 2020). In mammalians, methylation at the 5th carbon of cytosine (5-methylcytosine; 5-mC) is the most predominant DNA modification (Greenberg and Bourc’his, 2019). It is a post-replication modification that occurs when a methyl group is inserted in the cytosine residue of specific 5′- cytosine guanine-3′ dinucleotides (CpG sites), often clustered in CpG-rich regions (CpG islands) in the promoter region of a gene. This usually results in a reduced transcriptional activity of the gene (Lesch, 2011; Booij et al., 2013). By converse, when the methylated CpG residues are inside the gene, the result is the opposite: DNA transcription is stimulated and gene expression is increased (Klose and Bird, 2006). Besides 5-mC methylation, 5-hydroxymethylation (5-hmC) has recently attracted attention as it constitutes an intermediate in DNA demethylation process and is thought to play an active role in the regulation of gene expression as well. 5-hmC is particularly abundant in the central nervous system (CNS), compared to many other tissues, where it is 10-fold higher than in embryonic stem cells. In fact, both 5-mC and 5-hmC DNA methylation represent key players in brain plasticity since they are involved in several important processes, such as neural stem cell differentiation and environmental programming of molecular, hormonal and behavioral responses (Meaney and Szyf, 2005; Tognini et al., 2015).

### Neuroimaging: A Set of Techniques Commonly Used to Study Brain Development

Neuroimaging techniques comprise a large number of different powerful and non-invasive tools, which represent a unique window into the developing brain. In particular, Magnetic Resonance Imaging (MRI) can investigate both brain structural properties, such as the size of specific brain structures, and brain functioning, such as the cortical response to specific tasks/stimuli or its modulation in a resting condition.

Many different acquisition sequences can be used to investigate the structural properties of the brain. Among them, T1-weighted and T2-weighted are the most commonly used, as they are usually included in research and clinical protocols, and provide a good contrast between the gray matter and the other brain structures. Moreover, a wide collection of software has been developed to analyze T1-weighted and/or T2-weighted images in order to extract quantitative measures characterizing brain structure, such as volumes (Ashburner and Friston, 2000; Fischl et al., 2004; Avants et al., 2011), cortical thickness (Han et al., 2006; Das et al., 2009), and geometric measures (Ashburner and Friston, 2000; Schaer et al., 2008; Douet et al., 2014; Peruzzo et al., 2016). Diffusion Weighted Imaging (DWI) is another technique widely used to characterize the morphology of the white matter and its fiber bundle architecture. DWI images measure the signal changes associated to water diffusion in the nervous cells (Beaulieu et al., 1999; Vorona and Berman, 2015) allowing the characterization of white matter integrity (Mukherjee et al., 2008; Jensen and Helpern, 2010; Zhang et al., 2012) or structural connections among brain areas (Sporns et al., 2005). There is a wide assortment of other acquisition techniques that can be used to further investigate brain morphology, such as Fluid-attenuated inversion recovery (FLAIR) or double inversion recovery (DIR), although they are usually applied to specific brain structures or pathologies (Peruzzo et al., 2013; Mehrabian et al., 2019; Sone et al., 2020).

Brain functional properties can be investigated with functional-MRI (*f*-MRI) (Glover, 2011), Arterial Spin Labeling (ASL) (Detre et al., 2012), or MR spectroscopy (MRS) (Mandal, 2012). *f*-MRI is the most commonly used technique to measure brain activity *in vivo* by detecting signal changes associated with the Blood Oxygen Level Dependent (BOLD) effect. Functional studies can be either task based, i.e., they investigate changes in brain activity due to specific tasks or stimuli (Graham et al., 2015), or resting state (r*f*-MRI), i.e. they characterize the intrinsic spontaneous fluctuation in brain activity that are present even in rest condition (Gusnard and Raichle, 2001). An alternative method to obtain a functional related contrast can be the ASL technique (Detre et al., 2012), which provides a more direct measure of the perfusion changes induced by cortical activation than the BOLD effect. However, up to now ASL is mainly used to characterize brain perfusion rather than functional activity due to a lack of a standard acquisition set up and analysis framework. Differently from *f*-MRI and ASL, MRS is used to study metabolic changes and some attempts have been made to characterize brain function through the temporal fluctuation of metabolites (Gussew et al., 2010), with the advantage of assessing a specific aspect of cellular activity, but with the drawback of a partial and coarse brain coverage. Other techniques can be used to investigate brain activity without using an MRI scanner, such as electroencephalography (EEG) and functional near-infrared spectroscopy (*f*-NIRS). They are often characterized by a higher temporal resolution than fMRI, but also by a worse spatial resolution. EEG directly measures the electrical activity of the brain, while *f*-Near-infrared spectroscopy (NIRS) tracks the cortical hemodynamic response using near-infrared light sources, simultaneously recording cortical deoxygenated and oxygenated hemoglobin concentration changes with high temporal resolution providing high quality pictures (Cutini and Brigadoi, 2014).

### Neuroimaging and DNA Methylation: Rationale and Methodological Issues

Over the past decades, progress in both epigenomic and neuroimaging methods have separately contributed to important advances in the field (Poldrack and Farah, 2015). Notably, some points of contact between these approaches emerge, particularly regarding the mechanisms through which early life stress exposure affects the epigenome, as well as the developing brain. However, although studies on DNA methylation and brain vestiges of earl life stress exposures are rapidly accumulating (Provenzi et al., 2018), there is a paucity of studies assessing the association between DNA methylation and brain features in developmental age (Wheater et al., 2020). For this reason, there’s a growing interest in considering this kind of data together and in better outlining this innovative framework of research (Nikolova and Hariri, 2015). The application of DNA analysis to cognitive neuroscience seeks to identify molecular and neural predictors of human behavior (Lancaster et al., 2018) having potential consequences on several research fields including: neurodevelopment and neurodevelopmental disorders, psychopathology, post-traumatic stress disorder, addiction, aging and neurodegeneration, and socio-emotional processing (Wheater et al., 2020).

Since studies in this field are still in their dawn, several conceptual and methodological issues need to be addressed. First, it is important to recognize the reasons why we should be taking into account DNA methylation and neural correlates in a given study that investigates the effects of early life stress in a pediatric population. In fact, the lack of clear explanations of the interrelationship between these two kinds of variables may lead to the flawed assumption that the addition of DNA methylation and brain measures will improve the overall quality of a study *per se*. Second, to clearly understand the role of molecular and neural data in association with human behavior (Lancaster et al., 2018) it appears particularly relevant to consider the statistical approach (i.e., correlation, regression, moderation, or mediation models) used to test this relationship. Moreover, since this approach may provide new insights on potential pathways though which DNA methylation of candidate genes might contribute to long-term behavioral development by the modifications of specific brain structures (Wiers, 2012), we believed that it would be important to better understand the state of the art of this approach with respect to developmental early risk conditions. Wheater et al. (2020) reviewed studies using neuroimaging and DNA methylation approach across the life span. Nevertheless, their work aimed to give a comprehensive state of the art of the just mentioned approach. We believe that, when considering a pediatric population, it would also be relevant to take into account the antecedents of DNA methylation changes and consequently of brain features. As reported above, early life stress could have a key-role in explaining, at least partially, developmental plasticity. Thus, the current review aimed to outline the interplay between early life stress, DNA methylation and brain features. Here, we report studies that have applied neuroimaging and DNA methylation analysis in pediatric populations with a history of early life stress, investigating the statistical meanings of the variables included, focusing primarily on the relationship between DNA methylation and brain features. This is carried out with the purpose of clarifying the implications of the findings and providing the reader with a comprehensive conceptual and methodological framework.

## Materials and Methods

### Literature Search

The Preferred Reporting Items for Systematic Review and Meta-Analysis (PRISMA) guidelines (Moher et al., 2015) was adopted for the purposes of the present systematic review. A computer-based literature search was conducted on studies published up to March 2021 on the following database: PubMed, Scopus, Web of Science. The following search terms were used: (“neuroimage^∗^” OR “*f*MRI” OR “MRI” OR “DWI” OR “*f*NIRS” OR “dtMRI” OR “dMRI” OR “brain volume” OR “cortical thickness” OR “white matter”) AND (“DNA methylation”). In addition, a manual search of the references lists of relevant publication was carried out to identify further eligible papers.

### Selection

**FIGURE 1:**
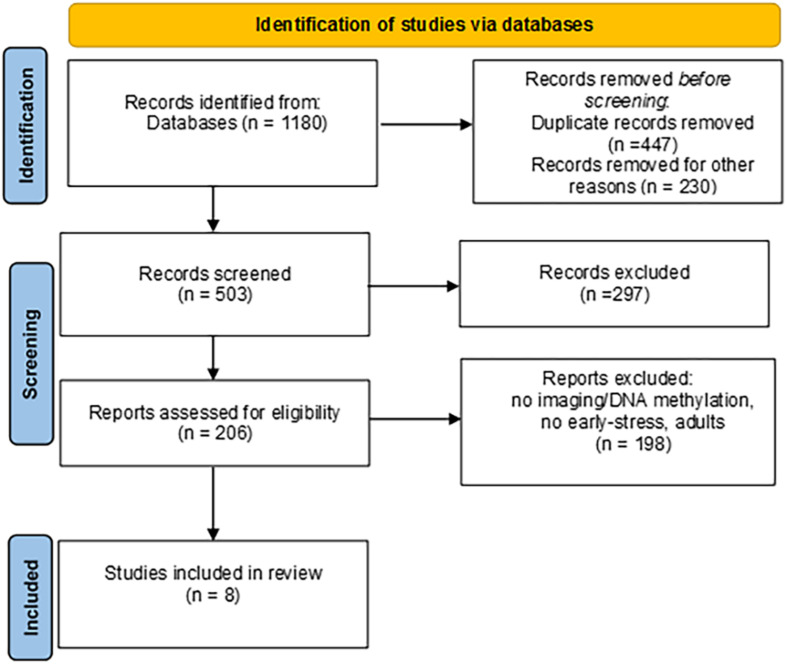
PRISMA flow diagram.

The papers were checked for duplicates. The remaining records were then filtered independently by two authors (ILCMW and EM) by reading titles, abstracts and the full articles. Disagreement was solved in conference. Exclusion criteria were: no DNA methylation; no neuroimaging; no early stress conditions; no developmental ages; genetic syndromes; theoretical papers; animal studies; non-English language papers. Procedural steps adopted are reported in Figure 1, together with record counts, duplicates, step-by-step criteria-guided screening and records obtained after each screening. Both authors initially performed an assessment of eligibility for inclusion and subsequently discussed their assessments to reach a final decision. We identified eight papers that meet the inclusion criteria.

### Data Abstracting

All the included records were reviewed and the following data were extracted: authors, year and country of publication, sample size, infants’ characteristics, early life stress, time/tissue and method of DNA methylation analysis, direction of methylation changes, time/brain areas and method of neuroimaging assessment, targeted brain areas, neurobehavioral outcomes, statistical model performed in order to test the relationship between DNA methylation and neuroimaging data.

### Quality Appraisal

The methodological quality of the included studies was assessed according to the Quality Assessment Tool for Quantitative Studies (Jackson and Waters, 2005). Two independent researchers (ILCMW and EM) coded sections from A to F according to the component rating scale criteria (1 = *strong*, 2 = *moderate*, 3 = *weak*). A summary score (see Table 1) between 1 and 3 was assigned to each paper according to the presence of no weak scores (1 = *strong*), one weak score (2 = *moderate*), two or more weak scores (3 = *weak*). A 94.6% of agreement was reached for the A-F components and the disagreement was solved through the senior author supervision (RM).

**TABLE 1:** Quality appraisal of the included studies.

Study	A	B	C	D	E	F	Final
Chen et al. (2015)	2	2	3	1	1	N.A.	2
Sparrow et al. (2016)	2	2	2	3	1	N.A	2
Harms et al. (2017)	3	2	2	2	1	3	2
Swartz et al. (2017)	2	2	1	2	1	2	1
Fumagalli et al. (2018)	2	2	1	2	1	3	2
Wrigglesworth et al. (2019)	2	2	2	2	1	3	2
Krol et al. (2019)	2	2	1	2	1	1	1
Fujisawa et al. (2019)	2	2	2	2	1	2	1

*Labels: A, Selection bias; B, Study design; C, Confounders; D, Blinding; E, Data collection methods; F, withdrawals and drop-out. Quality codes: 1, strong; 2, moderate; 3, weak. N.A., Not Available.*

## Results

A final pool of eight studies was obtained. All the studies included in this review highlighted the role of early life stress on DNA methylation of different genes and the variability in brain function (Harms et al., 2017; Swartz et al., 2017; Fumagalli et al., 2018; Krol et al., 2019; Wrigglesworth et al., 2019) and structure (Chen et al., 2015; Sparrow et al., 2016; Fujisawa et al., 2019). Additionally, four studies examined possible pathways linking DNA methylation, neuroimaging data and their effects on behavior in different developmentally risk conditions, such as childhood maltreatment, preterm birth, less-than optimal caregiving and low socioeconomic conditions (Swartz et al., 2017; Fumagalli et al., 2018; Fujisawa et al., 2019; Krol et al., 2019). Data extracted from the included studies are summarized in Table 2.

**TABLE 2:** Summary of sample characteristics, epigenetic analyses, neuroimaging techniques, and neurobehavioral data.

Study; Country	Sample size; Age (M;SD)	Early life stress and measure	Tissue for methylation analysis	Targeted genes	Analysis of CpG site methylation	Analysis method	Epigenetics variations		Neuroimaging techniques	Significant Brain areas	Putative main functions	Neurobehavioral outcomes and measures
Chen et al. (2015); Singapore	237; 38 weeks	Antenatal maternal anxiety; STAI-Y2	Umbilical Cord sample	Genome-Wide methylation and *BDNF*	Single CpGs	Infinium Human Methylation 450 Bead Chip assay (Illumina)	BDNF methylation and Val66Met polymorphism	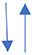	Structural MRI	Hippocampus and Amygdala	Learning and memory; Stress response and stress-related disorders	N.A.
Sparrow et al. (2016); United Kingdom	72; 38; 42 weeks	Preterm Birth	Saliva	Genome-Wide	Single CpGs	Pyrosequencing	*SLC7A5, SLC1A2, NPBWR1, APOL1, QPRT, LRG1, PRPH, GRIK5, TREM2, MCHR1*	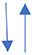	Diffusion MRI	right CST and Genu	Voluntary motor control and sensory modulation Coordination and complex problem solving	N.A
Harms et al. (2017); United States	5 54; T1: 11.2 years T2: 20.5 years	Early life stress; YLSI	Saliva	*FKBP5*	Single CpGs	NGS	FKBP5	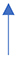	fMRI	dlPFR	Executive functions; successful response inhibition	N.A.
Swartz et al. (2017); United States	132; T1: 11–15 years T2: 13–18 years T3: 14–19 years	SES	Saliva	*SLC6A4*	Single CpGs	Pyrosequencing	SLC6A4	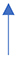	fMRI	Amygdala	Stress response; stress-related disorders	Depression symptoms; YSR Affective symptoms
Fumagalli et al. (2018); Italy	24; T1: 28–32 weeks T2: 35–40 weeks T3: 39–42 weeks T4: 12 months	Preterm Birth	Peripheral blood sample	*SLC6A4*	Single CpGs	NGS	SLC6A4	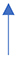	Structural MRI	ATL-LPL/R; ATL-MPL/R	Emotional regulation; social behavior	Socio-emotional development; GMDS Personal-Social scale
Wrigglesworth et al. (2019); Australia	33; 12.8; 0.3 years	Neighborhood disadvantages; IRSD	Saliva	*BDNF*	Single CpGs and average across multiple sites	EpiTYPER on Sequenom MassARRAY	BDNF	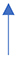	Structural MRI	PFC	Executive functions; emotional regulation; social behavior	N.A.
Krol et al. (2019); Australia	98; 147.97; 14.4 days	Maternal anxiety; IRI	Saliva	*OXTR*	Single CpGs	Pyrosequencing	OXTR	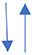	fNIRS	rIFC	Facial emotional processing; social behavior	Infant fearful temperament; IBQ-R
Fujisawa et al. (2019) Japan	85; 12.9; 2.6 years	Childhood maltreatment; CATS	Saliva	*OXTR*	Single CpGs and average across multiple sites	EpiTYPER on Sequenom MassARRAY	OXTR	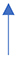	Structural MRI	OFC	Emotion and reward in decision making	Attachment style; IWMS

*N.A: Not Available; NGS: Next Generation Sequencing; STAI-Y2: State-Trait Anxiety Inventory; CST; corticospinal tract; YLSI: Youth Life Stress Interview; SES: Lower socioeconomic status; YSR Affective Symptoms: Youth Self Report (YSR) and the Affective Problems scores from the DSM-oriented scales; GMDS: The Griffith Mental Development Scales; IRSD: Index of Relative Socio-economic Disadvantage; IRI: Interpersonal reactivity index; IBQ-R: Revised Infant Behavior Questionnaire; CATS: Child Abuse and Trauma Scale; IWMS : Internal Working Model Scale.*

### Sample Characteristics

In the final pool of studies, developmental stages widely varied among the included studies: only two studied were conducted during the neonatal period (Chen et al., 2015; Sparrow et al., 2016), two in infancy (Fumagalli et al., 2018; Krol et al., 2019), one during childhood (Fujisawa et al., 2019), and three during adolescence with a history of early life stress in infancy or childhood (Harms et al., 2017; Swartz et al., 2017; Wrigglesworth et al., 2019). In the included studies different conditions were investigated: healthy neonates and infants exposed to a less-than optimal caregiving environment (i.e., maternal anxiety) (Chen et al., 2015; Krol et al., 2019), disadvantaged socioeconomic conditions (Swartz et al., 2017; Wrigglesworth et al., 2019), early adverse experiences, such as parental chronic medical or mental health problems, parental separation, unexpected death of a close family member, physically violent parents (Harms et al., 2017), preterm birth (Sparrow et al., 2016; Fumagalli et al., 2018), and a history of childhood maltreatment (Fujisawa et al., 2019).

### DNA Methylation and Neural Correlates

#### DNA Methylation

Based on the different aims of the final pool of studies included in this review, different genes were examined. Both genome-wide and candidate gene approach studies have been included. A genome-wide approach was used in two studies (Chen et al., 2015; Sparrow et al., 2016). As for the candidate gene approach studies, two papers focused on the stress-related *SLC6A4* gene, which codes for the serotonin transporter (Swartz et al., 2017; Fumagalli et al., 2018). The DNA methylation status of the promoter region of the oxytocin receptor gene (*OXTR*), which has been linked to a range of social and emotional processes, was investigated by two studies (Fujisawa et al., 2019; Krol et al., 2019). Two studies examined *BDNF* (Wrigglesworth et al., 2019), a neurotrophic factor that plays an important role in regulating neural development and plasticity. Finally, one study investigated the methylation level of the *FKBP5* gene (Harms et al., 2017), an important regulator of stress and glucocorticoid receptor sensitivity.

#### Neuroimaging Data

The studies included structural as well as functional research (Table 3). The images of the developing brain were collected using different neuroimaging techniques, such as structural MRI, diffusion MRI, *f*-MRI, and *f*-NIRS. From images of selected brain areas, studies extract information about volume, connectivity of white matter tracts, cortical thickness and functional activity. Specifically, structural MRI was used in four studies (Chen et al., 2015; Fumagalli et al., 2018; Fujisawa et al., 2019; Wrigglesworth et al., 2019). Volumetric features were considered in two of them (Chen et al., 2015; Fumagalli et al., 2018). In the study by Chen et al. (2015), the volume of the whole brain and of nine brain structures in both hemispheres (amygdala, caudate, cerebellum, globus pallidus, hippocampus, thalamus, total white and total gray matter and midbrain) were computed from structural T2W sequences (Chen et al., 2015). Fumagalli et al. (2018) quantified the volumes of four Region-Of-Interest (ROI: left-right hemisphere, lateral-media portion) in the anterior temporal lobes (ATL) using a combination of an automated segmentation and parcellation method on both T1W and T2W images, and a manual editing of the results (Fumagalli et al., 2018).

**TABLE 3:** Description of neuroimaging results.

Study	Field strength	Technique	Features	Software	Target ROI
Chen et al. (2015)	1.5T GE	Structural MRI	Volume	N.A	Total brain; total white matter (L/R); total gray matter (L/R); 7 subcortical strucures (L/R) (amygdala; caudate; cerebellum; globus pallidus; hippocampus; thalamus; mid brain)
Sparrow et al. (2016)	3T Siemens	Diffusion MRI	FA, MD, tract shape index (R)	FSL	Genu and splenium of corpus callosum, cingulum cingulate gyrus (L/R), CST (L/R), inferior longitudinal fasciculi (L/R)
Harms et al. (2017)	3T GE	Task f-MRI	Activation maps from an event recognition task	AFNI	Whole brain analysis
Swartz et al. (2017)	3T Siemens	Task f-MRI	Activation maps from an emotional face matching task	SPM8	Activation clusters in *a priori* selected ROIs (Amygdala)
Fumagalli et al. (2018)	3T Philips	Structural MRI	Volume	Automatic parcellation algorithm	Bilateral anterior temporal lobe lateral and medial parts (ATL-LPL, ATL-LPR, ATL-MPL, ATL-MPR)
Wrigglesworth et al. (2019)	3T Siemens	Structural MRI	Cortical thickness	FreeSurfer v5.3	22 *a priori* selected ROIs from Desikan-Killiany atlas
Krol et al. (2019)		fNIRS	Activation maps from an emotional face recognition task		Bilateral frontal and temporal lobes
Fujisawa et al. (2019)	3T GE	Structural MRI	Cortical GM local volume	SPM12 (VBM)	Voxel level in *a priori* selected ROIs (bilateral orbitofrontal cortex (OFC) and dorsal striatum)

*N.A: Not Available; FA: functional anisotropy; MD: mean diffusivity; CST: corticospinal tracts.*

Two studies focused on cortical features extracted from T1 weighted (T1W) images. One of them used cortical thickness measures of pre-selected ROIs in the prefrontal region (Wrigglesworth et al., 2019). Fujisawa et al. (2019) applied a voxel-based morphometry (VBM) approach in order to obtain local gray-matter volume measures in the bilateral orbitofrontal cortex (OFC) and dorsal striatum regions.

In one study, diffusion (dMRI) images were acquired to obtain white matter connectivity data from which eight major white matter fasciculi were segmented (i.e., genu and splenium of corpus callosum as well as left and right cingulum cingulate gyrus, corticospinal tracts inferior longitudinal fasciculi), and the mean values for the fractional anisotropy (FA), the mean diffusivity (MD), and tract shape index (*R*) were computed (Sparrow et al., 2016).

Three studies assessed brain activation patterns of a specific task performance. Two of them used an *f*-MRI technique (Harms et al., 2017; Swartz et al., 2017), while the latter exploited a *f-*NIRS acquisition (Krol et al., 2019). In the study by Harms et al. (2017), a “go no-go” task was used to assess the activation maps for an event recognition response, while Swartz et al. (2017) measured amygdala activation in a group of adolescents using an emotional face-matching task. Finally, Krol et al. (2019) examined the brain responses to emotional facial expressions in the frontal and temporal lobes, bilaterally.

### Statistical Models in Neuroimaging and DNA Methylation Studies

Although all studies have linked the DNA methylation status of specific genes and neural correlates, different statistical models were used in order to test the nature of this relationship in a pediatric population faced with early life stress conditions. In fact, while in some studies DNA methylation and neural correlates are simply associated to each other, in others they are treated either as predictors or as outcome variables, and in others again, they are investigated as moderators or mediators. Choosing one model rather than another is determined by different experimental assumptions, which in turn lead to a different interpretation of the findings. While correlational studies are not informative about the causal link and provide only the strength of the association between two variables, studies that apply linear regression models permit to describe the expected change in a dependent variable given one or more predictors. Differently, moderation and mediation analyses allow us to understand how one (or more) additional variables modulate the relationship between two (or more) variables. On one hand, identifying DNA methylation and/or neural correlates as moderators in the relationship with early life stress conditions and behavioral outcomes may provide insights into when certain relationships exist and when they may not. On the other hand, using DNA methylation and/or neural correlates as mediators may suggest the identity of the additional variables through which a main effect occurs.

**FIGURE 2:**
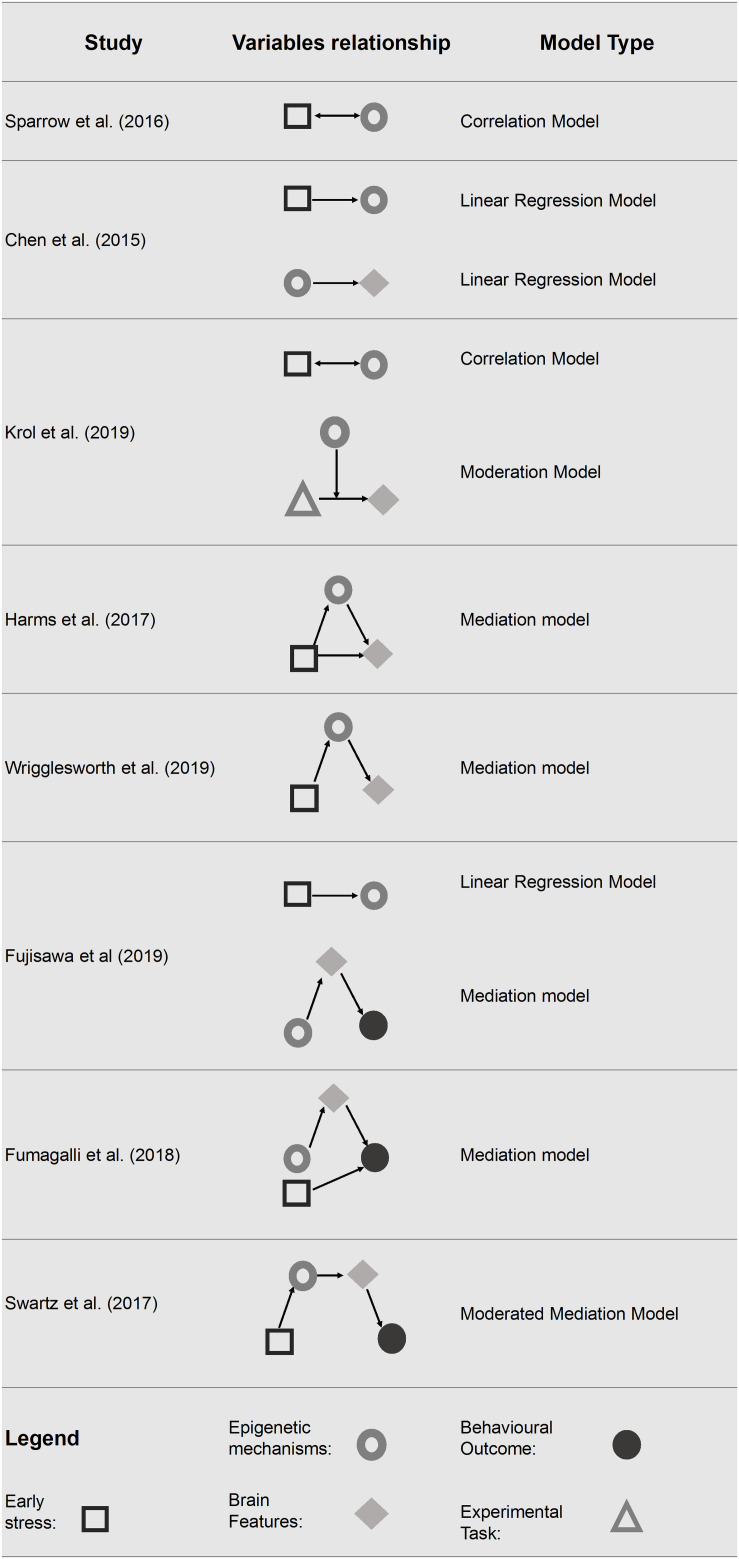
Statistical models tested in the final pool of studies. Only significant associations are reported.

Starting from this framework, we have synthesized the statistical approach used in the various studies to investigate, in pediatric populations faced with and early life stress conditions, the link between DNA methylation and neuroimaging data and, eventually, their relationship with behavioral outcomes. The statistical models used are summarized in Figure 2 and described below in order of complexity.

#### Correlation Model

The study conducted by Sparrow et al. (2016) examined whether preterm birth lead to DNA methylation changes and if these changes were associated with major white matter tracts, clinical risk factors (e.g., early nutrition factors and chorioamnionitis) and individual variables (e.g., gender).

The results reveal differences in DNA methylation in 25 gene bodies and 58 promoter regions of protein-coding genes in preterm infants compared to their full term counterparts. Notably, ten of these genes (i.e., *SLC7A5*, *SLC1A2*, *NPBWR1*, *APOL1*, *QPRT*, *LRG1*, *PRPH*, *GRIK5*, *TREM2*, and *MCHR1*) are known to influence neural cell function and to be associated with behavioral traits and neuropsychiatric diseases, such as mood disorders and schizophrenia (Shao and Vawter, 2008). Moreover, in order to test DNA methylation values related to white matter tracts parameters, a principal components analysis was performed. Ninety-five percent of the DNA methylation variance was explained by 23 principal components (PC). After correction for multiple testing, three significant associations remained: the 6th PC was significantly associated to right CST *R*; the 7th PC was significantly associated with both gender and duration of parenteral nutrition. Taken together, these results suggest that epigenetic variations may contribute to preterm infants’ cerebral phenotype.

#### Linear Regression Model

The study conducted by Chen et al. (2015) examined if the methylation level of *BDNF* predicts the volume of brain regions determined shortly after birth (Chen et al., 2015). The methylation level of different CpGs was examined separately for three groups of infants defined by their *BDNF* Val66Met genotype. Results have shown that nine of 18 brain variables had significantly disproportionate numbers of co-varying CpGs in at least one of the three *BDNF* Val66Met genotype groups. In particular, the right volume of amygdala showed disproportionately higher numbers of co-varying CpGs in the Met/Met group. By converse, the left hippocampal volume resulted in a disproportionately higher number of co-varying CpGs within the Val/Val genotypic group. Furthermore, infants’ Val66Met *BDNF* genotype seems to affects also the association between antenatal maternal anxiety and infants’ genome methylation status at birth; a greater influence of maternal anxiety on the neonatal epigenome among Met/Met compared to Val/Val carriers was found.

#### Moderation Model

The study conducted by Krol et al. (2019), examined whether the methylation status of *OXTR* was associated with neural responses to emotional expressions. The methylation level of *OXTR* was assessed at 5 months of age, while neural responses were recorded at 7 months of age using *f*-NIRS. During the *f-*NIRS procedure infants viewed happy, angry and fearful faces. Their results pointed out that the methylation level of *OXTR* (moderator) interacted with the emotional face processing (predictor) in explaining the right-inferior frontal cortex responses (outcome). Specifically, infants with higher *OXTR* methylation showed an enhanced neural response to anger and fear and an attenuated response to happiness in the right inferior frontal cortex (rIFC), a key structure in emotional processing.

Additionally, the study examined several bivariate associations. Among them, the one that is noteworthy for the purposes of the present review highlights how maternal anxiety is positively associated with *OXTR* methylation.

#### Mediation Model

Mediation models were tested in four of the included studies (Harms et al., 2017; Fumagalli et al., 2018; Fujisawa et al., 2019; Wrigglesworth et al., 2019). In particular, in two studies DNA methylation was considered as a mediator in the relationship between early life stress and neuroimaging data (Harms et al., 2017; Wrigglesworth et al., 2019). In two studies, neuroimaging data were used as a mediator in the relationship between DNA methylation and behavior (Fumagalli et al., 2018; Fujisawa et al., 2019). Harms et al. (2017) tested the methylation level of *FKBP5* as a mediator between early life stress exposure and prefrontal cortex activity. Specifically, the authors found that a high level of child stress exposure was associated with hypomethylation of *FKBP5* and reduced the prefrontal activation differentiation in response time, but not in accuracy, of error versus correct trials (Harms et al., 2017). Early life stress resulted in a less efficient recruitment of the dorsolateral prefrontal cortex (dlPFC), a key structure for successful response inhibition, suggesting that individuals with stressful childhood tend to require higher levels of engagement to suppress their predominant responses. This study showed that early life stress (predictor) has an effect on the activation of dlPFC (outcome) through the intervention of *FKBP5* methylation state (mediator). Similarly, Wrigglesworth et al. (2019) showed that higher neighborhood disadvantage (predictor) is associated with increased methylation level of *BDNF* (mediator) which, in turn, is negatively associated with cortical thickness (outcomes) in the bilateral lateral OFC and in the right medial OFC. Conversely, two of the studies included (Fumagalli et al., 2018; Fujisawa et al., 2019) identified brain volumes as a mediator considering the methylation level of targeted genes (respectively, *OXTR* and *SLC6A4*) and the socio-emotional competences in children. Fumagalli et al. (2018) showed that Neonatal Intensive Care Unit (NICU)-related stress is associated with greater delta methylation of *SLC6A4* from birth to NICU discharge in preterm infants. Moreover, the increased delta methylation level (predictor) was associated with reduced ATL volume (mediator), which, in turn, was associated with poor personal-social scale scores at 12 months (outcome) measured by the Griffith Mental Development Scales (GMDS) (Griffiths, 1979). Finally, NICU-related stress remained a significant predictor of GMDS Personal-Social score at 12 months of age. Similarly, Fujisawa et al. (2019) found that *OXTR* methylation level influenced the insecure attachment style trough brain morphology in children with a history of maltreatment. The gray-matter volume of the left OFC mediated the relationship between *OXTR* methylation (predictor) and insecure attachment style (outcome).

#### Moderated Mediation Model

Finally, one study performed a moderated mediation model in order to examine how DNA methylation and neuroimaging data may intervene in the relationship between low socio economic status and behavior (Swartz et al., 2017). Specifically, the model tested the indirect effect of families’ socioeconomic status on future changes in children and adolescents’ depressive symptoms showing that both methylation and neural correlates were mediators in this relationship. The results showed a negative indirect effect, pointing out that a lower socioeconomic status during childhood predicted increased *SLC6A4* methylation level and increased centromedial amygdala reactivity (mediators), which in turn predicted greater future depressive symptoms (outcome) in the group with a positive family history of depression (moderator).

## Discussion

The present work aims to provide the state of the art of the neuroimaging and DNA methylation field of research regarding a pediatric population faced with early life stress condition, focusing on the statistical role of DNA methylation and/or neural correlates in the relationship between early life stress and later-life cognitive and behavioral phenotypes. Regardless, the importance of distinguishing DNA methylation and/or neural correlates as predictors, outcomes, moderators or mediators is crucial to improve theory-driven research and to provide insights for future research in this field.

### State of the Art

Despite the growing interest for neuroimaging and DNA methylation approaches, there is still a paucity of studies in this field regarding pediatric populations exposed to early life stress. This relative lack of studies could explain the methodological heterogeneity in the included papers. First, the study samples included a wide range of ages, from newborns to late adolescents. However, despite the early period of life being a unique window for investigating early brain development (Suppiej et al., 2017), studies regarding the neonatal period are still a minority (Chen et al., 2015; Sparrow et al., 2016). Second, while 50% of the studies used a longitudinal design (Harms et al., 2017; Swartz et al., 2017; Krol et al., 2019; Wrigglesworth et al., 2019), only one started from early developmental stages (Fumagalli et al., 2018). Third, seven out of eight studies did not include a control group, which significantly reduces the robustness of their findings. Moreover, while all included studies have considered the role of early life stress events in the relationship between DNA methylation and brain maturation, only a sub-set (*n* = 4) of them also investigated behavioral outcomes in association with DNA methylation and neural correlates (Swartz et al., 2017; Fumagalli et al., 2018; Fujisawa et al., 2019; Krol et al., 2019).

An aspect that should be considered when dealing with neuroimaging studies is their intrinsic heterogeneity. The number of available MRI acquisition techniques, of sequence sets up, of image processing steps, of available software and of measurable features make each study virtually unique, as each of the mentioned aspects has an impact in the final measures. The cortical gray matter can be investigated measuring its volume, area, thickness or local deformation with respect to a template, while the activation maps derived from an *f*MRI study strongly depend on the implemented task and control conditions. The selection of the appropriate neuroimaging feature/phenotype is crucial to correctly answer the clinical questions in a neuroimaging study and becomes even more critical for the success of a neuroimaging-genetic study (Anderson et al., 2010; Shadia and Pernet, 2019). Furthermore, MRI derived quantitative measures are influenced by the acquisition sequence, the analysis pipeline and the selected software (Klein et al., 2009; Bowring et al., 2019) in a way that is independent from the selected feature (Kazemi and Noorizadeh, 2014; Tustison et al., 2014). These effects are usually accounted for using a control group or condition, whose measures are used to assess the data variability not due to the tested pathology or condition. Nonetheless, the identification of a control group/condition is not always straightforward in the epigenetic context. Finally, when combining image-derived measures with genetic ones in the statistical analyses the multiple comparison problem must be considered (Bennett et al., 2009). The multiple comparison problem arises when multiple hypotheses are tested on non-independent datasets (e.g., when multiple models are tested on the same neuroimaging data) or when the same hypothesis is tested in several datasets (e.g., when a given model is tested in each brain voxel independently). With the common significance threshold set to *p* < 0.05 each test has a 5% chance to produce a false positive result. Increasing the number of comparisons will increase the probability to get false positive results, situation that is exaggerated in the neuroimaging context, where thousands of spatially correlated voxels are usually considered (Bennett et al., 2011). Different approaches have been proposed in the literature to address such a problem (Worsley, 1996; Nichols and Holmes, 2001; Genovese et al., 2002), but a standard correction method has not been identified yet, as all of them provide a different tradeoff between sensibility to small effect sizes and robustness to type I errors. *A priori* assumptions on the expected features and/or brain regions to be considered in the study can significantly reduce the amount of comparisons performed in the statistical analysis and limit the multiple comparison problems. On the other hand, the neuroimaging and DNA methylation approach is quite new and few studies can be used to formulate *a priori* hypotheses.

### Model the Complex Relationships Between DNA Methylation and Neural Correlates

In an attempt to further improve our knowledge on how environmental experiences could be embedded in developmental processes, epigenetic mechanisms and neural correlates seem to be meaningful steps in the biological route between individual experiences and later-life behavioral phenotypes. Interestingly, despite the heterogeneity of the studies included here, these two factors are described as significantly related in all of the studies included and these results are in line with those obtained in adult research (Wheater et al., 2020). While one study treated DNA methylation and neural correlates as simply associated (Sparrow et al., 2016), other studies were able to test more complex models considering also behavioral outcomes (Harms et al., 2017; Swartz et al., 2017; Fumagalli et al., 2018; Krol et al., 2019; Wrigglesworth et al., 2019). These more complex models require additional considerations. First, findings reported in the current review suggest that DNA methylation always mediates the relationship between early life stress and neural correlates. Specifically, in the studies conducted by Harms et al. (2017) and Wrigglesworth et al. (2019) the relationship between early life stress, brain structures and functions was, at least partially, explained by *FBBP5* and *BDNF* methylation status, respectively. Second, in studies including behavioral outcomes, neural correlates mediate the relationship between DNA methylation and behavioral phenotypes. For example, *OXTR* (Fujisawa et al., 2019) and *SLC6A4* gene methylation (Fumagalli et al., 2018) were found to be related to altered brain volumes, which in turn predicted altered attachment style and socio-emotional competences in children, respectively. Therefore, while some studies focused on the “environment × DNA methylation × neural correlates” relationship, others focused on “DNA methylation × neural correlates × behavioral phenotypes,” thus considering only a part of the complex relationship between environmental experiences and behavioral phenotypes. Interestingly, only one study looks at the full picture, adding a more complex and complete point of view (Swartz et al., 2017). In this study, both *SLC6A4* methylation levels and changes in amygdala reactivity mediated the relationship between early life stress and depressive symptoms in children and adolescents. Thus, it is plausible to consider a consequentiality: adverse early life stress seems to cause DNA methylation changes, which in turn lead to brain modifications that finally could result in less-than optimal later-life behavioral phenotypes. In conclusion, although the role of DNA methylation and neural correlates seem to depend on the study design and statistical models considered, their contribution in explaining the relationship between early life stress and later-life outcomes seems to be meaningful and significant.

### Future Directions

Although the neuroimaging and DNA methylation approach is quickly gaining in popularity because of its great potential to advance our knowledge on developmental plasticity (Lancaster et al., 2018), future research would benefit from at least five salient directions. First, a considerable body of research indicates that epigenetic mechanisms are also sensible to early life positive experiences such as maternal sensitivity (Barry et al., 2008; Conradt et al., 2016). Similarly, brain maturation and organization seem to be sensible to early positive experiences (e.g., neuroprotective care, skin-to-skin contact) as well (Scher et al., 2009; Milgrom et al., 2010). Despite this, neuroimaging and DNA methylation studies in pediatric populations so far have focused only on risk factors (Graham et al., 2015; Montirosso et al., 2016). For this reason, it would be important to implement research in neuroimaging and DNA methylation examining the effects of protective factors on developmental plasticity.

Second, in order to study the long-term effects of risk and protective factors, a longitudinal experimental design is required (Lester et al., 2012). Future research using the neuroimaging DNA methylation approach should further focus on early life phases and continuously follow-up infants over later development stages in order to provide relevant insights for the biochemical and neurological underpinnings of behavioral and cognitive development in a pediatric population.

Third, it is still unclear which brain aspects are more involved in epigenetic mechanisms. So far, neuroimaging and DNA methylation studies in pediatric populations have each focused on a single acquisition technique or imaging-derived feature. Further studies comparing multiple MRI techniques and image-derived features are required to highlight not only the brain aspects more sensible to the DNA methylation impact, but also the causality aspects among them.

Fourth, as can also be seen from our results, studies on a small number of subjects do not allow the application of complex statistical models, as they require more subjects to provide stable and statistically robust results. Future studies in this field should therefore include a large number of participants in order to provide stronger and more generalized results.

Fifth, as DNA methylation markers in human subjects can usually only be obtained from peripheral tissues (e.g., blood and saliva), multi-dimensionally integrating data from peripheral markers of epigenetic regulation with CNS measures will strengthen research findings and help explaining, at least partially, the effects of environmental conditions on development and behavior in different developmental stages (Provenzi et al., 2019). In this regard, it should be mentioned that recent studies highlight the fact that DNA methylation patterns obtained from peripheral tissues appear to be generally equivalent between each other and to methylation patterns from brain tissues (Gregory et al., 2009; Perkeybile et al., 2018; Braun et al., 2019). Candidate gene studies suggested partial concordance between methylation measured in peripheral blood cells and umbilical cord blood cells in non-clinical populations (Braun et al., 2019) and strong correlations between methylation patterns obtained from saliva samples with those revealed from blood and brain cells (Gregory et al., 2009; Perkeybile et al., 2018; Krol et al., 2019; Puglia et al., 2020). In addition, a recent genome-wide research revealed robust correlations between saliva-brain (*r* = 0.90), blood–brain (*r* = 0.86) and buccal–brain (*r* = 0.85) methylation patterns (Braun et al., 2019).

To date, all studies that investigate the association between early life stress, DNA methylation and neuroimaging data considered 5-mC modifications only. Future research in the field should consider other DNA methylation modifications, such as 5-hmC. Moreover, a novel DNA adenine modification, N(6)-methyladenine (6-mA), has been recently found in mammalian cells (Fu et al., 2015). In mouse brain, for example, 6-mA levels has been found to be significantly elevated in response to environmental stress (Yao et al., 2017). Genes carrying stress-induced 6-mA changes significantly overlap with loci know to be associated with neuropsychiatric disorders such as autism spectrum disorder, depression and schizophrenia (Basu et al., 2009; Jia et al., 2010; Hyde et al., 2016). Therefore, 6-mA methylation should be considered as an intriguing candidate and thus examined in future neuroimaging and DNA methylation studies.

It should be noted that, in the final pool of studies, only two of them investigated DNA methylation through a genome-wide approach (Chen et al., 2015; Sparrow et al., 2016). Further research using genome-wide approaches are encouraged since they would allow to assess, among other things, the so-called *epigenetic risk profile* in a pediatric population (Chen et al., 2020). Finally, recent studies in the field took into account another epigenetic mechanism, namely microRNAs (Wang et al., 2020; He et al., 2021). Thus, it would be relevant to widen the investigation of the field including this epigenetic mechanism. Nevertheless, further research is encouraged to improve interdisciplinary approaches, such as neuroimaging and DNA methylation analysis, also creating synergy between different work groups.

## Conclusion

Developmental plasticity can be explained at multiple levels of analysis starting from the expression of the genetic code to individual observable behaviors. Since there are multiple steps in the biological route between genetic expression and behavior, neural correlates represent an intermediate factor that can provide additional valuable information about the development of and susceptibility to less-than-optimal phenotypes. Hence, neuroimaging and DNA methylation studies could shed light on how early individual experiences influence later-life phenotypes identifying predictors of dysfunctional developmental trajectories and epigenetic risk profile. Furthermore, data provided by the neuroimaging and DNA methylation approach embody the possibility to be used as biomarkers. In conclusion, this interdisciplinary approach could also provide stronger evidence, new insights and an important contribution to clinical activity.

## Data Availability Statement

The original contributions presented in the study are included in the article, further inquiries can be directed to the corresponding author.

## Author Contributions

RM and EM conceived and planned this manuscript. ILCMW carried out the search and revision of the literature and drafted the study. RM, EM, DP, RG, and SB reviewed and edited the writing. All authors revised the article critically for important intellectual content, commented on and approved the final manuscript and are accountable for all aspects of the work.

## Conflict of Interest

The authors declare that the research was conducted in the absence of any commercial or financial relationships that could be construed as a potential conflict of interest.
